# Impaired contractile function of the supraspinatus in the acute period following a rotator cuff tear

**DOI:** 10.1186/s12891-017-1789-5

**Published:** 2017-11-09

**Authors:** Ana P. Valencia, Shama R. Iyer, Espen E. Spangenburg, Mohit N. Gilotra, Richard M. Lovering

**Affiliations:** 1 Department of Orthopaedics, University of Maryland School of Medicine, AHB, Rm 540, 100 Penn St., Baltimore, MD 21201 USA; 2 Department of Kinesiology, University of Maryland School of Public Health, College Park, USA; 3 Department of Physiology, East Carolina Diabetes and Obesity Institute, Brody School of Medicine, East Carolina University, Greenville, USA

**Keywords:** Contractility, Muscle force, Rat, Eccentric injury, Neuromuscular junction, Collagen organization

## Abstract

**Background:**

Rotator cuff (RTC) tears are a common clinical problem resulting in adverse changes to the muscle, but there is limited information comparing histopathology to contractile function. This study assessed supraspinatus force and susceptibility to injury in the rat model of RTC tear, and compared these functional changes to histopathology of the muscle.

**Methods:**

Unilateral RTC tears were induced in male rats via tenotomy of the supraspinatus and infraspinatus. Maximal tetanic force and susceptibility to injury of the supraspinatus muscle were measured in vivo at day 2 and day 15 after tenotomy. Supraspinatus muscles were weighed and harvested for histologic analysis of the neuromuscular junction (NMJ), intramuscular lipid, and collagen.

**Results:**

Tenotomy resulted in eventual atrophy and weakness. Despite no loss in muscle mass at day 2 there was a 30% reduction in contractile force, and a decrease in NMJ continuity and size. Reduced force persisted at day 15, a time point when muscle atrophy was evident but NMJ morphology was restored. At day 15, torn muscles had decreased collagen-packing density and were also more susceptible to contraction-induced injury.

**Conclusion:**

Muscle size and histopathology are not direct indicators of overall RTC contractile health. Changes in NMJ morphology and collagen organization were associated with changes in contractile function and thus may play a role in response to injury. Although our findings are limited to the acute phase after a RTC tear, the most salient finding is that RTC tenotomy results in increased susceptibility to injury of the supraspinatus.

## Background

Rotator cuff (RTC) tears, particularly in the supraspinatus muscle, are a common orthopedic problem resulting in shoulder dysfunction and can result in disability [17, 41, 73, 76]. Despite substantial biologic tendon healing after a RTC repair, persistent problems include high re-tear rates and long-term functional deficits of the muscle-tendon unit that may persist even in the absence of a recurrent tendon tear [6].

In RTC tears, loss of tendon continuity is clearly the initial, paramount problem, but associated changes in the muscle are a major obstacle to full recovery. Large RTC tears can lead to irreversible muscle atrophy and fatty infiltration, especially in older patients [22, 39]. Muscle weakness can result in gleno-humeral instability and poor shoulder function [29, 70, 72, 78], but it is unclear how RTC tendon tears specifically impact strength of the RTC muscles. Much of the available data has been ascertained from studies on animals, which provide control over many variables (i.e. age, gender, history, etc.) and other advantages, such as a means to use identical injuries to study underlying mechanisms.

Previous work has suggested that the RTC muscles respond differently to injury from muscles in the hind limb [13], and that damaged RTC muscles have fewer satellite cells [31] with decreased proliferative capacity [46], all of which may help explain the poor outcomes observed after RTC tears compared to other muscle-tendon tears [25, 27, 75]. To the best of our knowledge, susceptibility to eccentric contraction-induced injury of torn RTC muscles has never been assessed, even though eccentric movement of the RTC is necessary for activities of daily living [51], and is recommended for shoulder rehabilitation [12, 32, 33, 80]. The overall aim of this work was to assess contractile function in the rat supraspinatus after a two-tendon RTC tear, and to compare such changes to biological markers such as atrophy, NMJ morphology, lipid content, and fibrosis. A second aim was to assess supraspinatus muscle susceptibility to eccentric injury after RTC tear. Such information on contractility and susceptibility to injury could help with decision making in the period leading up to repair and post-repair rehabilitation.

## Methods

All protocols were approved by the University of Maryland Institutional Animal Care & Use Committee. We used male rats (Sprague-Dawley, body weight 242 ± 11 g, Charles River Laboratories, Germantown, MD) at approximately 3 months of age. Rats were randomly assigned to three groups (Control, 2D, or 15D). Twenty rats underwent tenotomy 15 days (15D, *N* = 10) or 2 days (2D, N = 10) prior to muscle testing, and ten rats were used as weight-matched controls (CTRL, N = 10). Rats from each group were tested on the same day. Before each experiment, the animal was anesthetized (~ 4-5% isoflurane in an induction chamber, then ~ 2% isoflurane via a nosecone for maintenance) using a precision vaporizer (cat # 91103, Vet Equip, Inc., Pleasanton, CA). During the procedure, the animal was kept warm by use of a heat lamp. To avoid possible findings in histology, protein analysis, or imaging that might be due to muscle testing and/or eccentric injury, weight-matched rats (CTRL, 2D, and 15D, *N* = 6 each group) that did not undergo contractile testing were used.

### Tenotomy

Since the histopathology of the rat supraspinatus better mimics the human condition of RTC tear when the supraspinatus and infraspinatus tendons are cut [24, 36, 43, 61], both of these tendons were surgically released. Unilateral dual tenotomy of the supraspinatus and infraspinatus tendons were performed after induction of anesthesia. After shaving and cleaning the skin, a small longitudinal incision was made over the acromion and deltoid. The deltoid muscle was split to expose the superior aspect of the RTC. The supraspinatus and infraspinatus tendons were transected as distally as possible, both to mimic the typical location of a tear and to provide sufficient tendon for attachment to the load cell for testing at later time points. Incisions were closed using sterile Vicryl 4.0 silk suture (Johnson & Johnson, New Brunswick, NJ). All animals were monitored until recovery from the inhalation anesthesia, and buprenorpophine was administered (0.05 mg/kg) subcutaneously as needed.

### In vivo contractile function and susceptibility to injury

Contractile function of the supraspinatus muscle was measured in vivo as described previously [74]. Briefly, in the anesthetized animal, the scapula was immobilized in a custom designed rig as described previously [74] and the tendon of the supraspinatus muscle was released and attached to a load cell (FT03, Grass Instruments, Warwick, RI & QWLC-8 M, Honeywell, Morris Plains, NJ). The suprascapular nerve was stimulated via subcutaneous needle electrodes (36BTP, Jari Electrode Supply, Gilroy, CA) placed at the suprascapular notch. Single twitches (1 ms, S48 square pulse stimulator, Grass Instruments, West Warwick, RI) were applied at different muscle lengths to determine the optimal length (resting length, *L*_o_). At *L*_o_, a force-frequency plot was obtained by progressively increasing the frequency of pulses during a 200 ms pulse train.

For muscle injury, a custom program on commercial software (Labview version 8.5, National Instruments, Austin, TX) was used to synchronize contractile activation and the onset of forced lengthening. A stepper motor (model T8904, NMB Technologies, Chatsworth, CA) was used to induce muscle lengthening. Injury resulted from 30 forced lengthening contractions superimposed onto maximal isometric contractions spaced 0.5 min apart (CTRL *N* = 5, 2D *N* = 4, and 15D N = 4). The moment arm of the supraspinatus relative to the axis of rotation was ~3.7 mm, a 30^°^ angular displacement represents a strain approximating 15% L_0_ of the supraspinatus muscle, which is within the physiological range of supraspinatus lengthening. Maximal isometric force was obtained after 2 min rest and it was compared to the maximal isometric force recorded before injury protocol.

### Assessment of NMJ morphology

Supraspinatus muscles were dissected and stored in 4% paraformaldehyde until stained with α-bungarotoxin (α-BTX) conjugated to Alexa-488 (Molecular Probes B13423, Eugene, OR). A total of 80 NMJs were imaged (30 CTRL, 25 2D, 25 15D) and analyzed as described previously [54–56]. Labeling was performed on tissue whole mounts harvested from the mid-belly, the point at which the nerve enters the muscle. Whole mounts were sampled from at least 3 animals in each group. Digital images of NMJs from whole mount tissue preparations were obtained with a Zeiss 510 confocal laser-scanning microscope with pinhole set at 1.0 Airy unit. A maximum intensity flat plane projection was made from Z-stacked images in ImageJ software (NIH) to account for the depth of the NMJ. Only NMJs in a complete *en face* view were selected for analysis. After background was subtracted and noise despeckled, a Gaussian Blur filter with σ = 2.00 was applied. Binary images were then generated from which total area and total perimeter were quantified using tracing tools for the total NMJ endplate. Dispersion index (DI) was calculated as total stained area / total area * 100, describing NMJ density. To quantify continuity and branching of the NMJ, binary images were skeletonized and histograms describing the connectivity for each pixel were generated as previously described [56]. Histogram bins correspond to the number of neighboring pixels for each pixel. One neighbor implies a terminal pixel, two neighbors imply a pixel along a single branch, and 3 or more neighbors indicate that a pixel exists at a branch node. Thus, discontinuities (terminal pixel) or branching (3+ neighbors) may be quantified within the motor endplate [38].

### Lipid droplet staining

Muscles (> 3 sections per muscle) were sectioned in the mid-belly at a thickness of 10 μm and were stained with BODIPY-493/503 (Invitrogen, Carlsbad, CA) at 1:200 dilution for 1 hour to identify neutral lipid in muscle (*N* = 6 per group). Sections were mounted in Vectashield. Sections were visualized using a confocal microscope (Zeiss 510), and fluorescence of ~600 muscle fibers per group was quantified using ImageJ software (NIH, Bethesda, MD) as previously described [48]. Briefly, the integrated density, mean gray value, and area were measured for individual muscle fibers (~100 myofibers per animal), along with several background readings. The fluorescence for each muscle fiber was calculated by the following equation: Integrated density – (area of muscle fiber × mean fluorescence of background readings) × 100.

### Western blotting

Samples (*N* = 4 per group) from the mid-belly of supraspinatus muscles (50 mg) were homogenized in tissue-TEK lysis buffer (Invitrogen), and protein concentration was measured using BCA protein assay (Thermo Fisher Scientific). In a 4-15% gradient gel, 20 μg of protein were loaded, and separated proteins were transferred to a nitrocellulose membrane. Membranes were stained with Ponceau red (Sigma) to confirm successful transfer of protein and equal loading of lanes. Membranes were then blocked in 5% milk and incubated overnight in primary Anti-Ubiquitin antibody (Sigma, cat number U0508) at a 1:500 dilution. Membranes were visualized after incubation with HRP-conjugated goat antibodies and ECL substrate (Thermo Fisher Scientific). Bands were quantified using ImageJ software and normalized to total protein.

### Sirius red staining

Sections were stained for 1 h with Sirius red (0.1% Direct Red saturated in aqueous picric acid, Sigma), and rinsed with acidified water (5% acetic acid). Samples (*N* = 6 per group) from the mid-belly of the muscle (>3 sections per muscle) were mounted and imaged under brightfield microscopy followed by polarized light microscopy (Nikon). Pictures were taken under the same conditions and exposure time. Birefringent collagen was then analyzed as previously described [65]. Briefly, we determined the number of pixels with 8-bit hue thresholds for red, orange, yellow, and green using ImageJ. The proportion of each hue was calculated by dividing the pixels for each hue to the sum of total colored pixels.

### MRI imaging

Small animal in vivo magnetic resonance imaging (MRI) was performed as described [49, 57, 71]. High-resolution dual-echo proton density and T2-weighted rapid acquisition relaxation-enhanced (RARE) MR images (TR/TEeff/NA, 1500.00 ms/12.94 ms/4) were on a 7 Tesla Bruker Biospec 7 T/30 MR system (Biospec 7 T/30; Bruker Biospin, Billerica, Massachusetts) with a four-channel phased array surface coil. T2-weighted images with and without fat-suppression were acquired for one animal at day 2 and day 15 after tenotomy to evaluate the fat content in supraspinatus muscle. Contralateral shoulder was used as a control. For ex vivo imaging, harvested supraspinatus muscles from each group were fixed in 4% paraformaldehyde, patted dry, and placed in a conical tube with Fluorinert FC-40 solution (Sigma). High-resolution T2-weighted RARE MR images (TR/TEeff/NA, 2500.00 ms/30 ms/1) with and without fat-suppression were acquired (2.5 h scan).

### Statistical analysis

Normality and homogeneity of variance were verified for all data before analysis (SigmaStat, San Rafael, CA). To evaluate potential differences between the three groups a One-Way ANOVA was used. Post-hoc Holm-Sidak test was performed to identify differences compared to the control group. Significance was set at *p* ˂ 0.05 and data are represented as mean ± standard deviation.

## Results

**Fig. 1:**
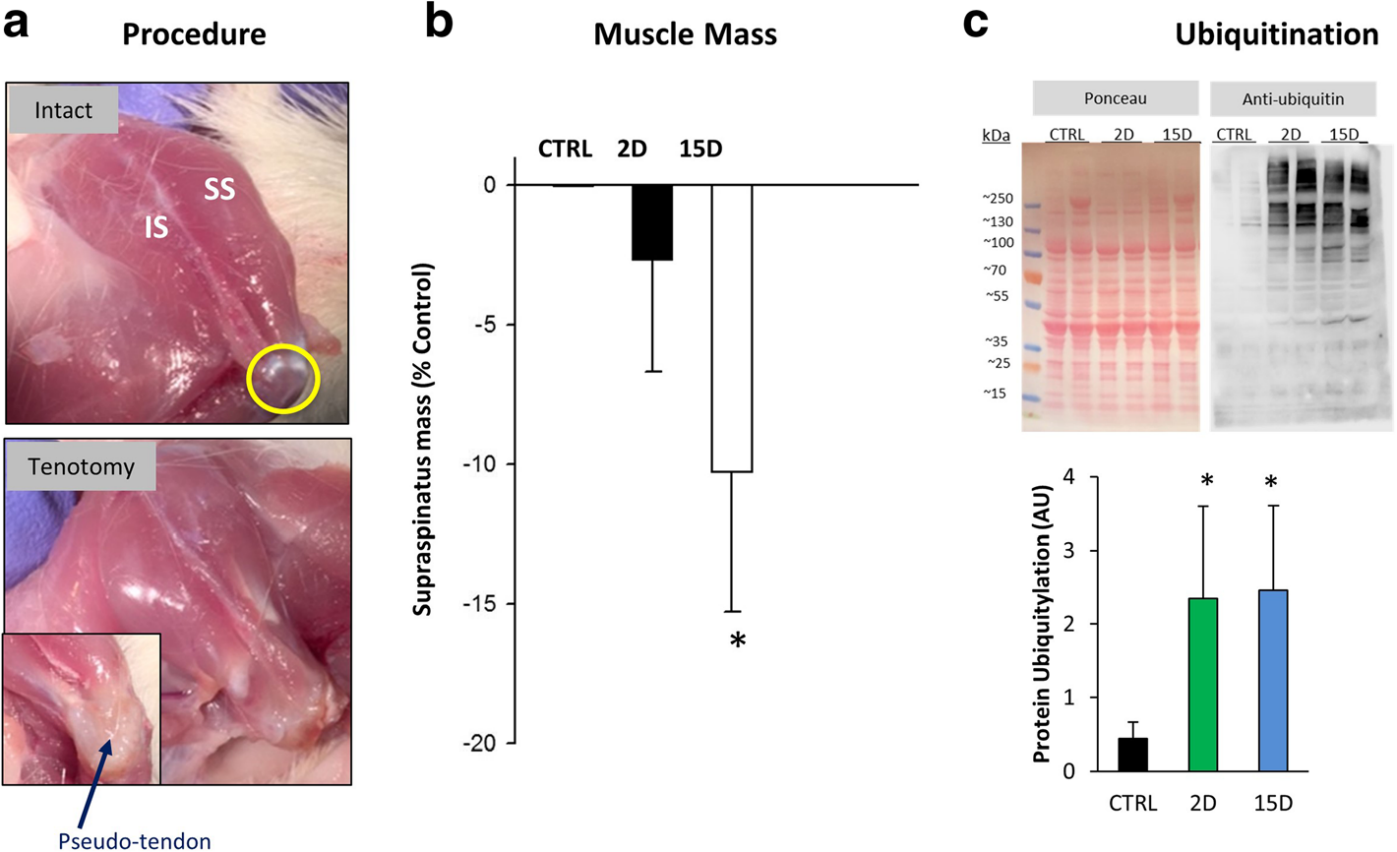
Supraspinatus tendon in a model of a RTC tear. **a** *Top panel* **.** Normal anatomy of the rat RTC (used as control) is shown, with the supraspinatus muscle (SS) and infraspinatus muscle (IS), including attachment of their tendons to the greater tubercle of the humerus (yellow circle). RTC tear was surgically induced by tenotomizing the supraspinatus and infraspinatus tendons. *Bottom panel* **.** After 2 days (2D), the RTC tear results in retraction of the tendons. Fifteen days after RTC tear (15D), the space between the muscle tendon and insertion site is filled by a fibrous-connective tissue (arrow) that reattaches the supraspinatus to the humeral head (inset). **b** As expected, supraspinatus muscle mass was slightly altered after tenotomy and significantly reduced by day 15. **c** Western blot analysis was used to detect ubiquitinated proteins in total protein extracts of supraspinatus muscles. Equal amounts of protein were loaded and confirmed with Ponceau and probed with anti-ubiquitin antibody. Total protein ubiquitination was upregulated at 2 and 15 days after tenotomy in supraspinatus muscle compared to control (CTRL). All data are presented as mean ± SD, *p* < 0.05. *, indicates statistical significance compared to control

Tendon transection resulted in supraspinatus muscle retraction of approximately 5 mm (Fig. 1a) by day 2, or almost 20% of resting muscle length in the rat supraspinatus [74]. There was no further change in muscle shortening over time, but the tendon scarred down by day 15, in such a way that the space between the tendon and insertion site was filled by a fibrous-connective tissue, forming an ill-defined “pseudo-tendon” that reattaches the muscle to the humeral head (Fig. 1a, inset) [4, 77]. As expected and shown by others [23, 43, 77], there was a loss of muscle mass 15 days after supraspinatus tenotomy (*P* = 0.04 Fig. 1b). The progressive decrease in muscle mass after tenotomy was preceded by increased conjugation of ubiquitin to muscle proteins in total cell lysate from muscles at day 2 (*P* = 0.01); and remained elevated at day 15 (*P* = 0.01; Fig. 1c), suggesting higher protein degradation via the ubiquitin-proteasome pathway [8].

**Fig. 2:**
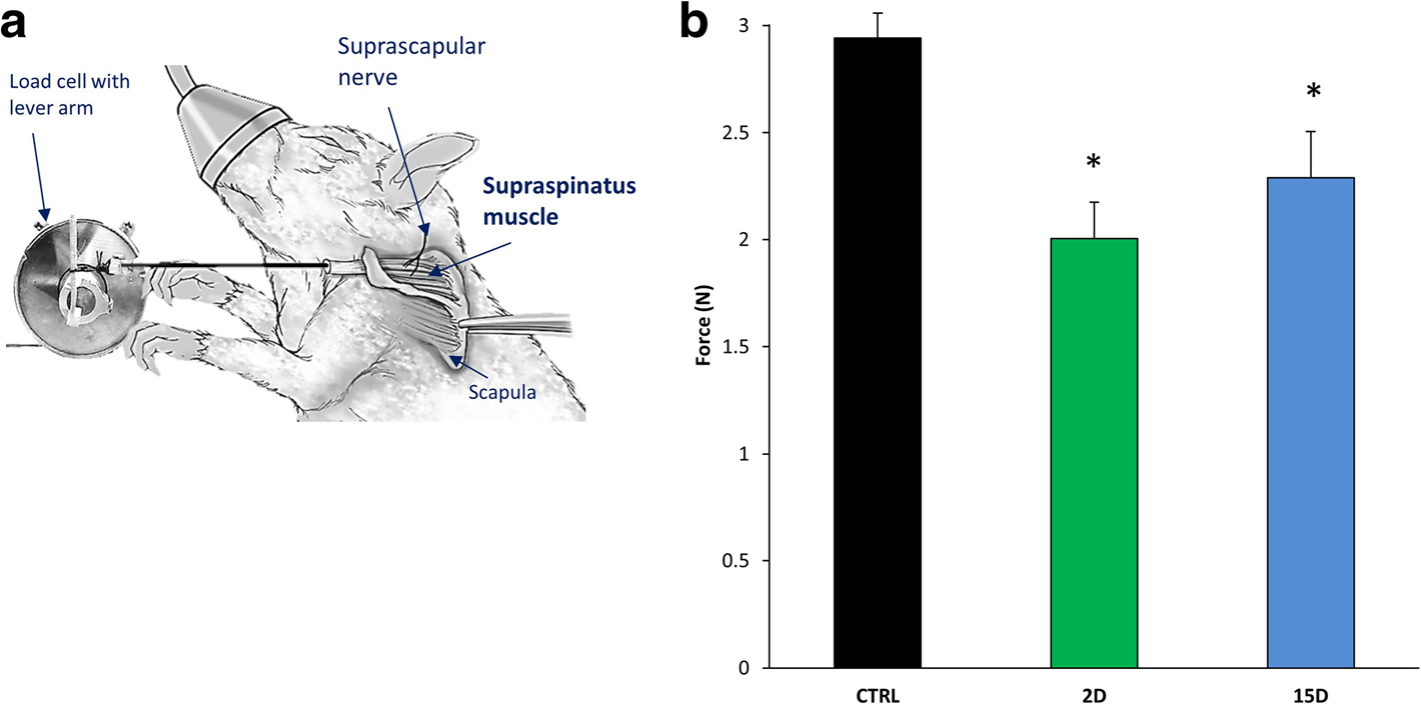
Maximal isometric force is lower in tenotomized supraspinatus at 2D and 15D. **a** Apparatus to measure muscle in vivo contractility and susceptibility to injury in the supraspinatus muscle. The insertion of the supraspinatus was released and the tendon tied to a load cell. The suprascapular nerve was stimulated via subcutaneous needle electrodes to activate the supraspinatus maximally. A series of maximal twitches was used to determine optimal length (L_o_) and the force-frequency relationship was determined to obtain maximal isometric force. **b** When compared to control, the mean of maximal isometric force per group was 30% lower at 2D and 20% lower at 15D. Maximal force was not different between the tenotomized groups. All data are presented as mean ± SD, *p* < 0.05. *, indicates statistical significance compared to control

**Fig. 3:**
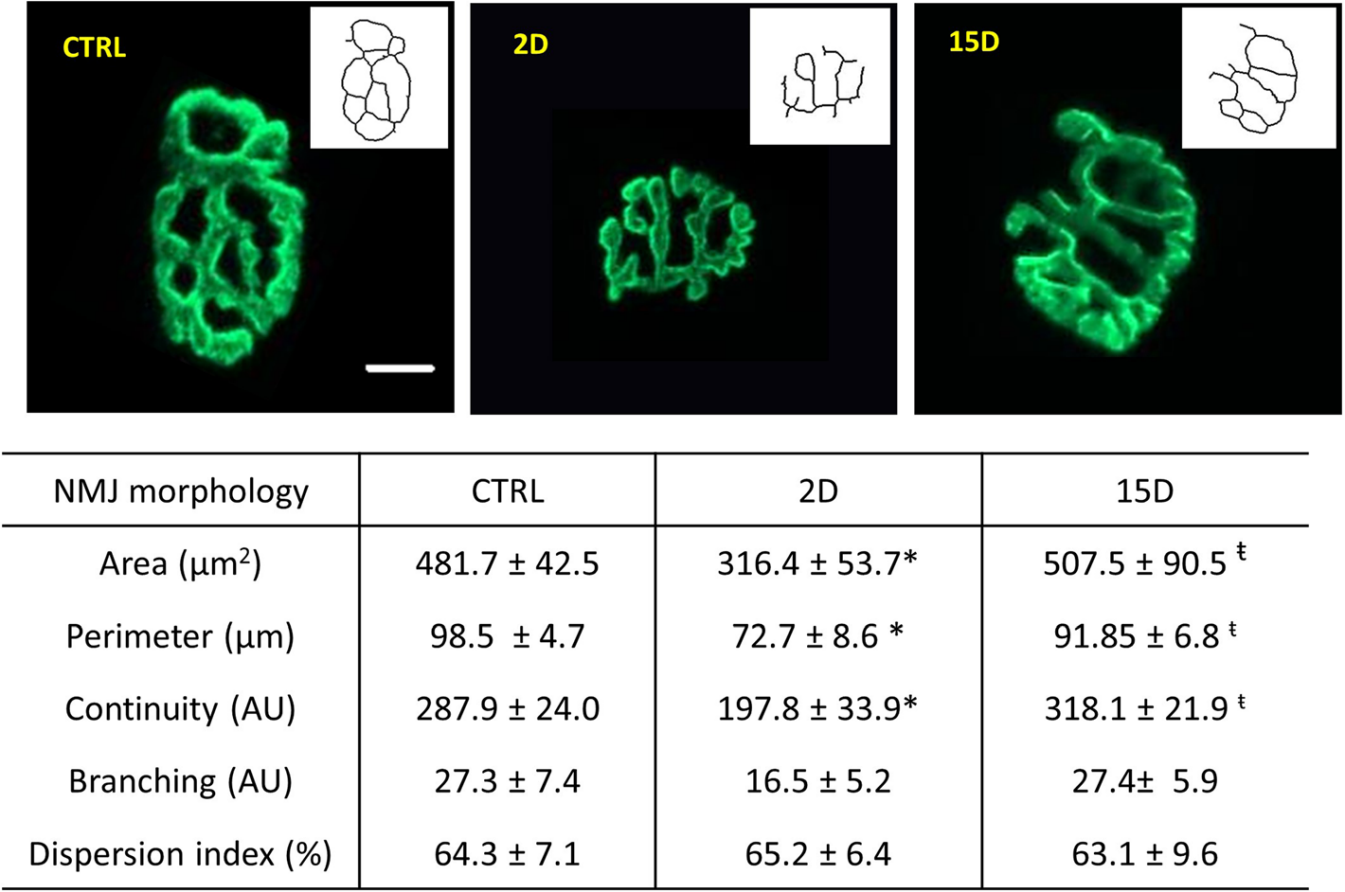
NMJ morphology is altered in tenotomized supraspinatus at 2D, but recovers at 15D. Neuromuscular junctions (NMJs) of at least three supraspinatus muscles per group were fluorescently stained with an acetylcholine receptor binding neurotoxin (α-Bungarotoxin, BTX, green) and imaged using confocal microscopy. Z-stacked images were analyzed and quantified using ImageJ software. Skeletonized images are shown in the white panel for each NMJ to further illustrate continuity and branching of NMJs. At 2D, NMJs were smaller and morphology was altered, as evidenced by decreased continuity of NMJ branches. No significant differences were seen in NMJ morphology compared to control at 15D. Scale bar represents 10 μm. All data are presented as mean ± SD, *p* < 0.05. *, indicates statistical significance compared to control, and ŧ indicates statistical significance compared to 2D

By day 15, there was a 10% decrease in muscle mass and a 20% reduction in muscle force compared to control (*P* = 0.007; Fig. 2, blue bar). However, at day 2 there was a 30% decline in isometric force (*P* = 0.0002; Fig. 2, green bar) despite no significant loss in muscle mass (*P* = 0.31; Fig. 1b). Our findings suggest that mechanisms beyond simple atrophy influence contractile force 2 days after tenotomy. Since there is a strong structure-function relationship for the NMJ, and its disruption likely results in altered excitation-contraction coupling [54], i.e. muscle activation, we assessed NMJ morphology. At the 2-day time point, NMJs exhibited significant reductions in area, perimeter, and altered continuity, compared to the control (*P* = 0.007) and 15-day group (*P* = 0.004; Fig. 3).

**Fig. 4:**
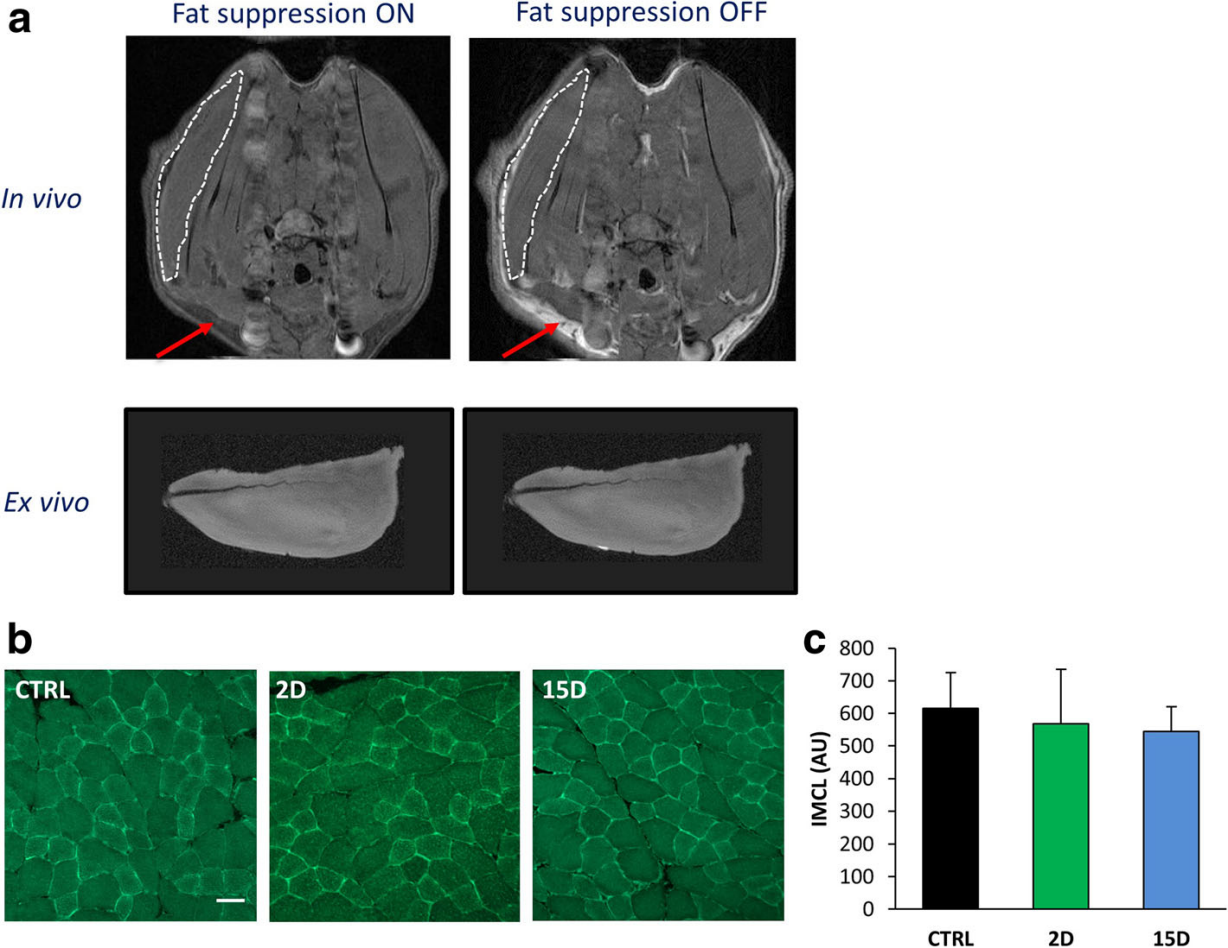
Lipid content is not altered in tenotomized supraspinatus at 2D or 15D. **a** Axial sections of in vivo magnetic resonance imaging (MRI) of a rat 15 days after unilateral RTC tear. When fat suppression is turned off, the white signal represents fat (note the obvious white signal from subcutaneous fat, red arrow). Fat was not detected in any axial sections in the torn supraspinatus (outlined by white dotted line). A longer (> 2 h), more detailed MRI scan of supraspinatus muscles ex vivo corroborated this finding. **b** Neutral lipids were stained in cross-sections from the mid-belly of the supraspinatus using Bodipy-493/503 and quantified (**c**) using ImageJ. No differences in intramyocellular lipid at 2D and 15D were found compared to controls. Scale bar represents 50 μm. All data are presented as mean ± SD, *p* < 0.05

We have used small animal magnetic resonance imaging (MRI) previously to assess the overall structure of hindlimb muscles [44, 45, 49, 53, 57, 79]. Here, we applied this modality in vivo and ex vivo to detect fat in the supraspinatus muscle. We compared T2-weighted images with fat-suppression to T2-weighted images without fat-suppression. Although the technique was effective to visualize subcutaneous fat (Fig. 4a, red arrows), *intramuscular* fat was not detected at any time point after RTC tear (not all time points shown). This was consistent with absence of any increases in intramyocellular lipid content with histological staining (*P* = 0.602; Fig. 4b-c).

**Fig. 5:**
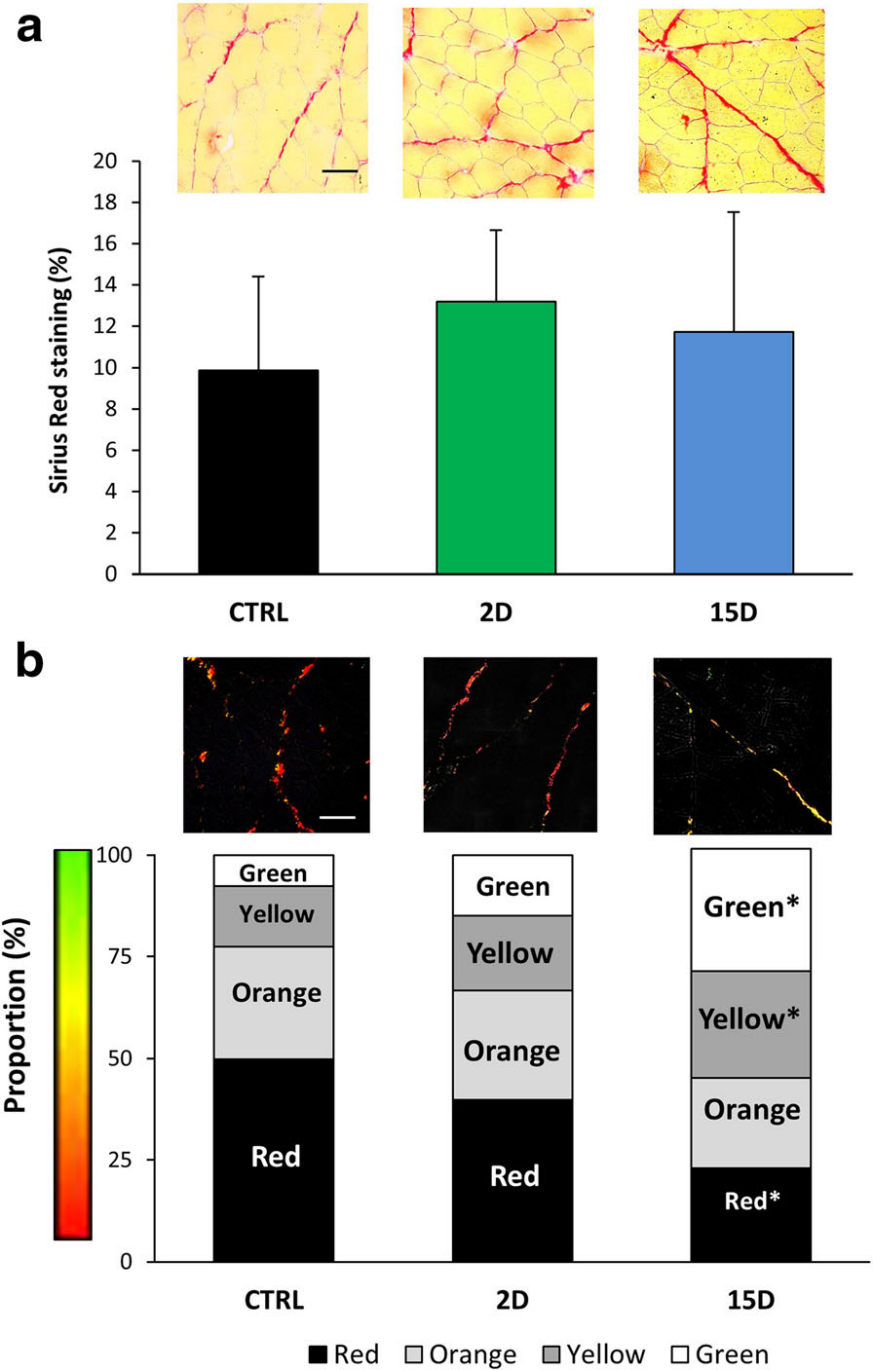
Collagen organization is altered in tenotomized supraspinatus at 15D. **a** Representative images from the mid-belly of muscle cross-sections stained with Picosirius red viewed under brightfield microscopy. Collagen content was visualized using Picosirius red staining, and quantified using a threshold on ImageJ to calculate the percentage of pixels stained per area. No differences in total collagen content are evident between groups. **b** Collagen organization was assessed using Picosirius red-stained sections viewed under polarized light. Red represents more densely packed collagen that is perpendicular to muscle fibers, and green represents loosely packed collagen that is parallel to the fibers. When analyzed as a proportion to total colored pixels, supraspinatus muscle at 15D has a lower proportion of red pixels and greater proportion of yellow and green pixels compared to control, indicating altered collagen organization (decreased collagen density, crosslinking, and thickness) at 15D after tear. No differences were evident at 2D compared to control. Scale bar represents 50 μm. All data are presented as mean ± SD, *p* < 0.05. *, indicates statistical significance compared to control

There are conflicting results regarding fibrosis in the rat supraspinatus after RTC tear, with some investigators reporting fibrosis [23, 43] while others do not [62, 68]. We did not find an increase in the percent area of interstitial collagen staining (*P* = 0.492; Fig. 5a). However, based on the birefringent properties of collagen stained with Picosirius red under polarized light [1, 50, 65] (see Methods), there was a change in collagen organization at day 15. Collagen birefringence in control muscles had a greater proportion of pixels closer to the red spectrum (*P* = 0.007; Fig. 5b) when analyzed as a proportion of total colored pixels. However, supraspinatus muscles at 15D had a greater proportion of yellow and green pixels compared to control (*P* = 0.017, *P* = 0.007) indicating altered collagen organization (i.e. reduced collagen packing density, thin collagen) [65]. No differences were evident at 2D compared to control.

**Fig. 6:**
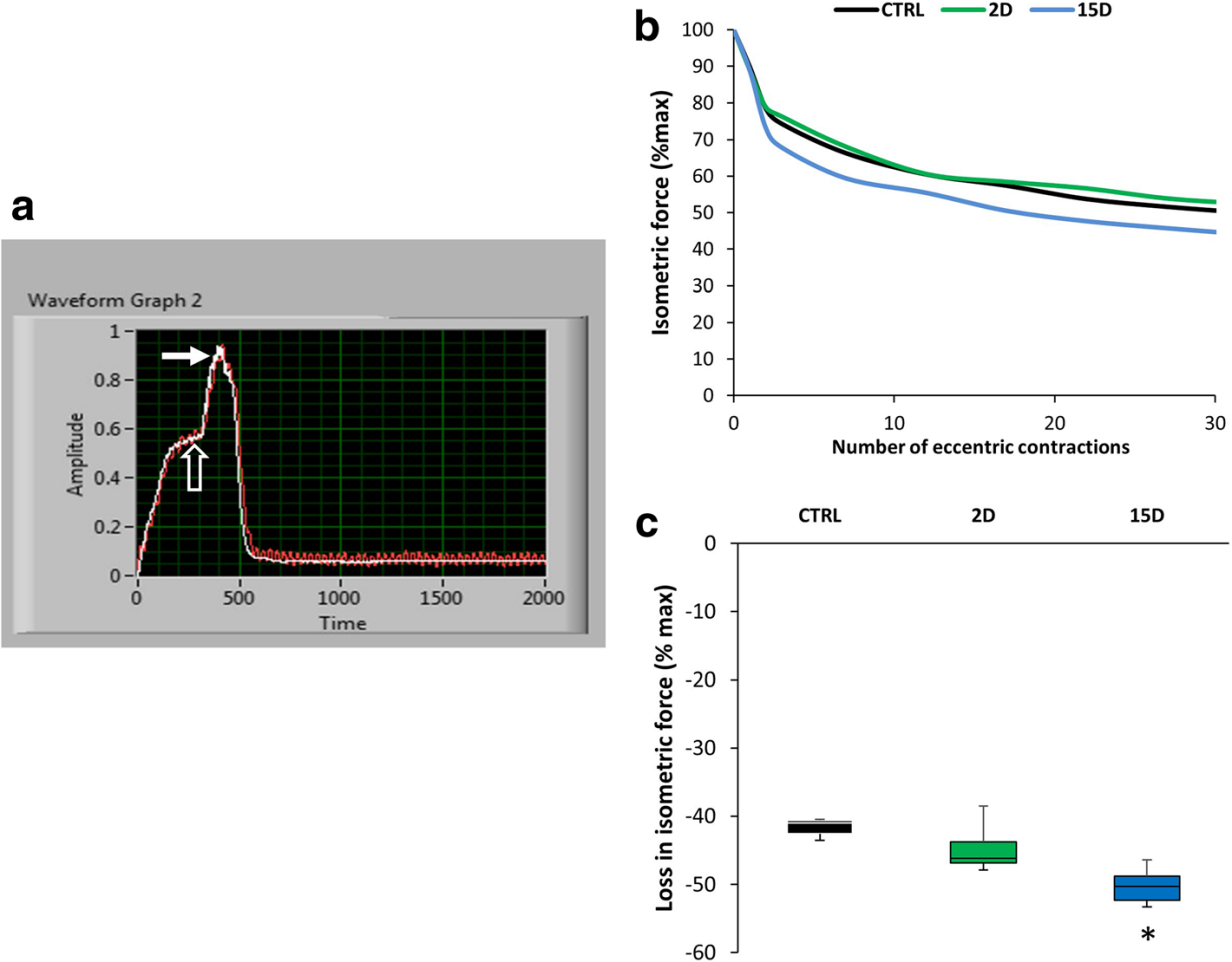
Tenotomized supraspinatus becomes more susceptible to injury at 15D but not at 2D. The same apparatus used to collect isometric force (Fig. 2) was also used to induce injury. The suprascapular nerve is used to stimulate the supraspinatus maximally while movement of the lever arm resulted in forced linear lengthening of the muscle (15% L_o_). **a** Representative screen shot showing force from a single eccentric contraction (closed arrow) superimposed onto a maximal isometric contraction (open arrow; y-axis in volts, which is converted to units of force based on calibration). **b** Representative rep-by-rep isometric force loss for one animal in each group throughout eccentric contraction protocol. **c** The mean loss of force for each group after the injury protocol. Despite an identical injury protocol, there is a greater drop in isometric force after injury at 15D (51.8 ± 2.5%) compared to control. All data are presented as mean ± SD, *p* < 0.05. *, indicates statistical significance compared to control

The screenshot in Fig. 6a shows an example of a lengthening contraction of the supraspinatus (closed arrow) superimposed onto a maximal isometric contraction (open arrow). We used a protocol of 30 eccentric contractions to the supraspinatus to induce injury and examined the loss in maximal isometric force. Fig. 6b illustrates the loss in isometric force after each eccentric contraction for a representative animal from each group. Supraspinatus muscles at 15D were more susceptible to injury evidenced by a greater drop in isometric force followed by a short recovery period after injury (*P* = 0.009; Fig. 6c). Absolute force before and after injury was 2.47 N ± 0.80 and 1.41 N ± 0.42 for control, 2.11 N ± 1.11 and 1.16 ± 0.59 for 2D, and 2.22 N ± 0.69 and 1.06 N ± 0.23 for 15D respectively.

## Discussion

RTC tears result in measurable histological changes to the RTC muscles [43, 61] but none of these indirect biological markers can account for the changes in contractile function [26, 66, 67]. Muscle contractile function is therefore considered the most valid and comprehensive measure of muscle health [10]. Our findings suggest that muscles become weaker and susceptible to injury after a simple tenotomy, even without direct trauma to the muscle fibers.

Given the high rate of poor outcomes after shoulder surgery, understanding the mechanisms leading to insufficient function is critical to develop effective treatments. Similar to other studies, we found a significant loss in muscle mass of RTC muscles 2 weeks following RTC tear [34, 42, 77]. While the ubiquitin-proteasome pathway is the main protein degradation pathway of skeletal muscle, previous studies show no changes in expression of key ubiquitin ligases, e.g. muscle RING-finger protein-1 (MuRF)1 and muscle atrophy F-box (MAFbx), after RTC tear [23, 42], as their expression can be transient as atrophy progresses [16]. Given our finding of acute increased protein ubiquitination (a downstream process of ubiquitin ligase expression) preceding significant loss in supraspinatus mass after tenotomy, the ubiquitin-proteasome system may play a more significant role in muscle atrophy induced by RTC tear than previously suggested [23, 34]. It is possible for ubiquitin ligases to be more active during the acute phase of injury, rather than later time points. Furthermore, additional ubiquitin ligases have been identified to regulate muscle mass [7] that have not been assessed in torn RTC muscles. We cannot rule out the possibility that the lack of changes in muscle mass at 2D was due to swelling, as inflammation and water content were not analyzed.

Although muscle atrophy could be a contributing factor to the decrease in contractile function, the initial drop in contractile force was evident before a significant decrease in muscle mass, paralleling the transient change of NMJ morphology. The NMJ has been implicated as a possible contributing factor to loss of contractile force [19, 20, 47, 59], and the notion of NMJ morphology changing after muscle mechanical strain is not new, but this is one of the very few studies to examine the NMJ after RTC tear. The gross morphology of NMJs has been examined qualitatively in a small sample of biopsies in the human supraspinatus [20], which classified acetylcholine receptor (AChR) staining along a range of morphologies (singlet dot, a doublet, a cluster, or a line) and concluded “the trend in innervation status is interpreted as leaving open the possibility that denervation plays a role in RTC injury pathophysiology” [20]. Here, we rigorously examined the NMJ using established methods [54–56] to provide precise, quantifiable measures of morphology. Others have reported no changes in the NMJ using an animal model of RTC tear [19], but that study was conducted in different species (rabbit) and only examined at one time point late after injury (3 months). It is possible that assessing the NMJ at such a late time point accounts for those negative findings, as turnover of AChRs has been reported on a timeline of days [3, 69]. We examined NMJ morphology after tenotomy at a time point when changes at the NMJ occur after injury [55, 63] and found changes during the period when muscle atrophy could not explain the loss in muscle force. We did not examine the nerve axon (i.e., the suprascapular nerve). With retraction of the severed muscles, the suprascapular nerve can be exposed to excessive tension at the suprascapular notch and/or spinoglenoid notch [64]. In animal models, the suprascapular nerve is sometimes cut intentionally, as the outcome from a simultaneous neurotomy-tenotomy better mimics fatty atrophy seen in patients. However, the severity of neurotomy does not allow for study of recovery of muscle contractile function after a tendon tear, and thus was not used in this study. It is possible that neither the nerve nor the NMJ are responsible for the loss in force at 2D as other possible explanations, such as disruption of contractile proteins, are not ruled out.

Fatty infiltration of muscles in patients with RTC tear involves the presence of adipocytes within the muscle (also known as intramuscular adipose tissue). Our findings agree with several studies showing that fatty infiltration after RTC tear is not substantial in the rat [43] compared to the levels seen in humans [5] or rabbits [60] after RTC tear. In addition to the formation of adipocytes in muscles, skeletal muscle fibers have the ability to store lipid in the form of small lipid droplets [9, 58]. While myofibers from patients with RTC tear also have an increase intramyocellular lipid [68], intramyocellular lipid did not increase after RTC tear in our rat model.

The extracellular matrix (ECM) contributes to muscle structure, transduction of mechanical force, remodeling, passive loading, and elasticity [40, 52], but excess accumulation of ECM in muscle (fibrosis) is common in pathological conditions [21, 40]. Fibrosis is evident after RTC tear in some studies [23, 43], but not others [62, 68]. Mechanical properties of tissue are not only affected by the amount of ECM, but also by the organization of ECM components, particularly collagen [2]. Collagen *content* has been measured after a RTC model in animals, but collagen *organization* has not. Genes involved in collagen turnover (i.e. matrix metalloproteinase and tissue inhibitor metalloproteinase) have an impact on collagen organization, and are upregulated in a rat model of RTC tear [14]. Using the birefringent properties of collagen stained with Picosirious red, we determined that collagen organization was altered 15 days after RTC tear [28, 65]. Decreased crosslinking of collagen is associated with increased collagen turnover, and increased crosslinking could contribute to muscle stiffness, both affecting the mechanical function of the muscle [2]. Although we do not show causality, we found altered collagen organization in the supraspinatus when it was also most susceptible to injury by eccentric contractions.

Eccentric injury commonly induces muscle inflammation and fibrosis, so the repair of a muscle that is apparently healthy, but susceptible to injury, could compound the dysfunction already induced by the RTC tear alone. For instance, one group found that muscle fibers become injured at the time of surgical tendon repair in a rat model of chronic RTC tear [15]. Although, it is currently unknown if susceptibility to injury is preventable, knowing the timeline of when the muscle is most susceptible to injury could help with decision making for optimal timing for repair, repair tension, and post-operative rehabilitation. It is possible that metabolic changes in the muscle come into play with an injury protocol, however allowing sufficient time between contractions after the protocol should have ruled out muscle fatigue alone as a factor.

A limitation in the rat in this study that prevented us from assessing contractile force at later time points is the spontaneous reattachment to the humerus via a pseudo-tendon. This means our findings are also limited to an acute period after tenotomy. While some investigators make no mention of adhesions or reattachment of the cut rotator cuff tendons in a rat model [24], others report that re-attachment of the supraspinatus tendon occurs spontaneously after tendon transection in rats at time points exceeding 2 weeks [4, 11, 77]. Spontaneous reattachment of the supraspinatus tendon is associated with recovery of muscle mass and collagen content [4, 77]. Efforts to avoid reattachment include removing the distal fragment of tendon [30, 43] or using a membrane [18, 24] to prevent spontaneous reattachment, while other studies make no mention of these deterrents [23, 24, 35–37]. We initially tried using membrane and even a polymer gel (not shown) after tenotomy to prevent spontaneous tendon reattachment. Such methods not only failed to prevent the tendon from scarring down, but also resulted in massive inflammation and an increase of variability in the data. Our experience suggests that removal of the tendon is likely to yield a result that best mimics the human condition of a RTC tear.

Additional limitations include the lack of sham surgery (skin incision and deltoid muscle split, but RTC remains preserved) and the limited window of follow-up mentioned above, making our results more relevant to the acute period after a RTC tear. The birefringent properties of collagen stained with Picosirius red has been previously used to assess collagen organization, but this indirect method is another potential limitation. Finally, the ubiquitin proteasome pathway needs to be more fully studied to determine which ubiquitin ligases are responsible for atrophy during the acute and long-term phases after a RTC tear.

Fiber type composition affects the speed of a muscle contraction, but less so the specific tension (force per unit area). Force depends not only on the size and number of the fibers, but also on muscle architecture. The maximal specific tension of skeletal muscle is considered relatively constant, but we did not assess architectural change or the contractile machinery (actin and myosin content) within fibers. Such variables could also contribute to the findings.

## Conclusions

This study describes histological and functional changes in the supraspinatus muscle in a rat model of RTC tear. The most salient findings of this work are the apparent dissociations between atrophy and muscle force soon after a RTC tear, as well as the finding that the supraspinatus becomes more susceptible to contraction-induced injury. Still, knowing when a torn RTC muscle is most susceptible to injury could be useful in surgical and rehabilitation planning, but additional work is needed to elucidate the specific timing and significance of this increased susceptibility to injury in patients.

## Abbreviations

15D: 15 days after tenotomy
2D: 2 days after tenotomy
AChR: Acetylcholine receptor
CTRL: Control
DI: Dispersion index
ECL: Enhanced chemiluminescence agent
ECM: Extracellular matrix
HRP: Horseradish peroxidase
MRI: Magnetic resonance imaging
NA: Number of averages
NMJ: Neuromuscular junction
RARE: Rapid acquisition relaxation-enhanced
RTC: Rotator cuff
T2: Spin-spin relaxation time
TEeff: Effective echo time
TR: Repetition time
α-BTX: α-bungarotoxin
